# Effect of Thrombolytics on Delayed Reperfusion After Incomplete Thrombectomy: Target Trial Emulation

**DOI:** 10.1212/WNL.0000000000213641

**Published:** 2025-04-28

**Authors:** Adnan Mujanovic, Vignan Yogendrakumar, Felix C. Ng, Thomas Gattringer, Bettina Lara Serrallach, Thomas R. Meinel, Leonid Churilov, Oliver Nistl, Shaokai Zheng, Peter J. Mitchell, Nawaf Yassi, Mark W. Parsons, Gagan Jyoti Sharma, Hannes A. Deutschmann, Geoffrey Alan Donnan, Marcel Arnold, Fabiano Cavalcante, Eike I. Piechowiak, Timothy John Kleinig, David Julian Seiffge, Stephen M. Davis, Tomas Dobrocky, Jan Gralla, Markus Kneihsl, Urs Fischer, Bruce C.V. Campbell, Johannes Kaesmacher

**Affiliations:** 1 Department of Diagnostic and Interventional Neuroradiology, University Hospital Bern Inselspital, University of Bern, Switzerland;; 2 Department of Neurology, Royal Melbourne Hospital and Melbourne Brain Centre, University of Melbourne, Melbourne, Victoria, Australia;; 3 Division of Neurology, The Ottawa Hospital and Ottawa Hospital Research Institute, University of Ottawa, Ontario, Canada;; 4 Department of Neurology, Austin Health, Heidelberg, Victoria, Australia;; 5 Division of Neuroradiology, Vascular and Interventional Radiology, Department of Radiology, University Hospital Graz, Medical University of Graz, Austria;; 6 Department of Neurology, University Hospital Graz, Medical University of Graz, Austria;; 7 Department of Neurology, University Hospital Bern Inselspital, University of Bern, Switzerland;; 8 Melbourne Medical School, The Royal Melbourne Hospital, University of Melbourne, Parkville, Victoria, Australia;; 9 ARTORG Center for Biomedical Engineering Research, University of Bern, Switzerland;; 10 Department of Radiology, The Royal Melbourne Hospital, University of Melbourne, Parkville, Victoria, Australia;; 11 Population Health and Immunity Division, The Walter and Eliza Hall Institute of Medical Research, Parkville, Victoria, Australia;; 12 Department of Neurology, Liverpool Hospital, University of New South Wales, Sydney, Australia;; 13 Department of Radiology and Nuclear Medicine, Amsterdam University Medical Centers, University of Amsterdam, Amsterdam Neuroscience, the Netherlands;; 14 Department of Neurology, Royal Adelaide Hospital, South Australia, Australia;; 15 Diagnostic and Interventional Neuroradiology, CIC-IT 1415, CHRU de Tours, France; and; 16 Le Studium Loire Valley Institute for Advanced Studies, Orléans, France.

## Abstract

**Background and Objectives:**

More than half of the endovascularly treated ischemic stroke patients with incomplete reperfusion (expanded Thrombolysis in Cerebral Infarction [eTICI] <3) show delayed reperfusion (DR) on 24-hour perfusion imaging, which is associated with favorable clinical outcome. The effect of intravenous thrombolysis (IVT) on the rates of DR remains unclear. This study aimed to assess the treatment effect of IVT on the occurrence of DR.

**Methods:**

Pooled data from 3 randomized controlled trials (EXTEND-IA and EXTEND-IA TNK parts 1 and 2) and 2 comprehensive stroke centers (University Hospitals Graz and Bern) were analyzed. Only patients with a final reperfusion score of eTICI 2a-2c and available perfusion imaging at follow-up of 24 ± 12 hours were included. The primary outcome was the presence of DR on 24-hour follow-up CT/MRI perfusion imaging, defined as the absence of any focal perfusion deficit on perfusion imaging, despite incomplete reperfusion on the final angiography series during thrombectomy. For the secondary analysis, we explored the association between the primary outcome (DR) and the time elapsed between start of IVT and the end of an intervention. To address confounding in observational data, we performed a target trial emulation.

**Results:**

Of 832 included patients with eTICI 2a-2c (median age 74 years, 49% female), 511 (61%) had DR. There was an independent treatment effect of IVT on DR (standardized risk ratio [sRR] 1.1, 95% CI 1.0–1.3; standardized risk difference [sRD] 8.2%, 95% CI 0.2%–16.1%), after adjusting for age, sex, atrial fibrillation, number of device passes, collateral score, and eTICI. Among those patients who have received IVT (n = 524/832, 63%), when adjusting for the aforementioned covariates, there was a causal effect of shorter time between administration of thrombolytics and end of the intervention on DR (sRR 0.93%, 95% CI 0.87–0.98; sRD −5.2%; 95% CI −9.1% to −1.3%, per hour increase).

**Discussion:**

Exposure to thrombolytics showed independent treatment effect on the occurrence of DR among patients with incomplete reperfusion after thrombectomy who undergo perfusion imaging at the 24-hour follow-up. The effect of thrombolytics on DR was observed if there was a high chance of therapeutic concentrations of thrombolytics at the time point when the proximal vessel was recanalized, but distal occlusions persisted and/or occurred.

**Classification of Evidence:**

This study is rated Class III because it is a nonrandomized study and there are substantial differences in baseline characteristics of the treatment groups.

## Introduction

While endovascular thrombectomy (EVT) has improved reperfusion rates for acute ischemic stroke patients, a considerable proportion of patients still do not achieve complete reperfusion (defined as the extended Thrombolysis in Cerebral Infarction, eTICI 3).^1^ All guidelines advise on achieving complete reperfusion whenever possible^2,3^ because patients with eTICI 3 have the highest likelihood of favorable clinical outcomes.^4^ Recent analyses have shown that 60% of patients with incomplete reperfusion (<eTICI 3) after EVT achieve complete delayed reperfusion (DR) within 24 hours. These patients have been found to have comparable outcomes with those who achieve eTICI 3 reperfusion immediately during the intervention.^5-7^

The effect of IV thrombolysis (IVT) on the rates of DR remains unclear. Previous data suggested that patients with running IVT on flow restoration have improved outcomes,^8,9^ with the prevailing hypothesis being that this association is due to clearance of persisting vessel occlusions or microemboli. This is also supported by a post hoc analysis of the Chemical Optimization of Cerebral Embolectomy (CHOICE) trial, suggesting less persistent hypoperfusion in patients receiving thrombolytics after EVT.^10^ However, current studies do not suggest an association between IVT and the occurrence of DR.^11,12^ These studies were single-center observations with limited sample sizes and retrospective study designs that focused exclusively on the use of IV alteplase. The potential effect of IV tenecteplase on DR remains completely unknown.

In this study, we evaluated the treatment effect of IV alteplase and tenecteplase on DR using target trial emulation. Target trial emulation mimics the design and conditions of a randomized controlled trial in a setting of observational research, to reduce bias and confounding.^13^ It is particularly useful when trials are infeasible or unethical.^13^ By emulating a target trial, the primary question of this study was as follows: among acute stroke patients with incomplete reperfusion (eTICI<3) immediately after mechanical thrombectomy and perfusion imaging at 24-hour follow-up, does thrombolysis increase the likelihood of DR compared with no thrombolysis?

## Methods

### Standard Protocol Approvals, Registrations, and Patient Consents

This study is a pooled international multicenter analysis of patients who underwent perfusion imaging at 24-hour follow-up after EVT. The study is compliant with the Declaration of Helsinki and reported according to the Strengthening the Reporting of Observational Studies in Epidemiology guidelines. Data pooling from all individual trials, the prospective observational study, and the stroke registry included in this analysis received ethics approval from their respective institutions. Signed informed consent for data use was obtained from all the included participants or their legal representatives. This study has been approved by the ethics committee (KEK ID 231/2014, 2020-01696, 2023-00892, 30–254 ex 17/18, HREC/11/MH/247, 2014.262).

### Data Set Description and Target Trial Inclusion Criteria

We pooled data from 3 randomized controlled trials, 1 prospective observational study, and 1 prospective stroke registry. These include “Extending the Time for Thrombolysis in Emergency Neurological Deficits–Intra-Arterial” (EXTEND-IA, clinicaltrials.gov, NCT01492725)^14^; “Tenecteplase versus Alteplase before Endovascular Therapy for Ischemic Stroke” (EXTEND-IA TNK parts 1 and 2, clinicaltrials.gov, NCT02388061 and NCT03340493, respectively)^15,16^; data from a prospective observational study from the University Hospital Graz (clinicaltrials.gov, NCT05273216); and prospectively collected stroke registry data from the University Hospital Bern.^5,17^ Methodology of these studies has been described previously.^5,14-17^ In short, the EXTEND-IA trial was a randomized trial assessing safety and efficacy of endovascular therapy plus thrombolytics (alteplase 0.90 mg/kg) vs thrombolytics alone.^14^ For this analysis, we used data from the endovascular therapy plus thrombolytics arm only. The EXTEND-IA TNK trials evaluated the safety and effectiveness of tenecteplase in the context of large vessel occlusion stroke. In EXTEND-IA TNK 1, patients were randomized to tenecteplase 0.25 mg/kg or alteplase 0.90 mg/kg,^15^ whereas in EXTEND-IA TNK 2, patients were randomized to tenecteplase 0.25 mg/kg or tenecteplase 0.40 mg/kg.^16^ The University Hospital Graz is a tertiary-level hospital serving as a reference center for stroke patients in southern Austria. The observational study from Graz prospectively included consecutive patients from 2018 to 2022 who underwent endovascular therapy with perfusion imaging on follow-up.^17^ The University Hospital Bern serves as a referral center for stroke patients in central Switzerland with a thrombectomy service available 24/7. The stroke registry from Bern prospectively captured all stroke patients admitted and/or treated between 2015 and 2020.^5^ These specific trials and studies were included because of the availability of perfusion imaging on either CT or MRI at 24 hours after the intervention, which was required to evaluate the evolution of incomplete reperfusion (in “Primary Outcome”). Moreover, the protocols of these trials and description of the studies have been previously published, providing reassurance of data quality and completeness—both critical for reliable target trial emulation.^13^ Only patients fulfilling the following trial inclusion criteria were considered for this analysis: (1) older than 18 years, (2) underwent EVT, (3) final reperfusion score expanded Treatment in Cerebral Infarction (eTICI) 2a-2c, (4) available perfusion imaging at follow-up of 24 ± 12 hours.

### Imaging Data and Ratings

All imaging sequences used for the analysis (eTable 1) were transferred through a secure cloud-based server, and all imaging outcomes (mentioned further) were centrally evaluated by an adjudicated core laboratory blinded to treatment allocation (with or without IVT). Collateral grading was performed using the American Society of Intervention and Therapeutic Neuroradiology and Society of Interventional Radiology (ASITN/SIR) Collateral Flow Grading System on preinterventional angiography images. Reperfusion success was evaluated with the eTICI scale on the final angiography series. Final angiography series were also screened for the presence of vasospasm, and all the cases with vasospasm, accompanied by slow/incomplete blood flow, were excluded from the final analysis. Intervention-to-follow-up time was defined as the duration between the final angiography series and the first follow-up imaging series.

### Primary Outcome

**Figure 1:**
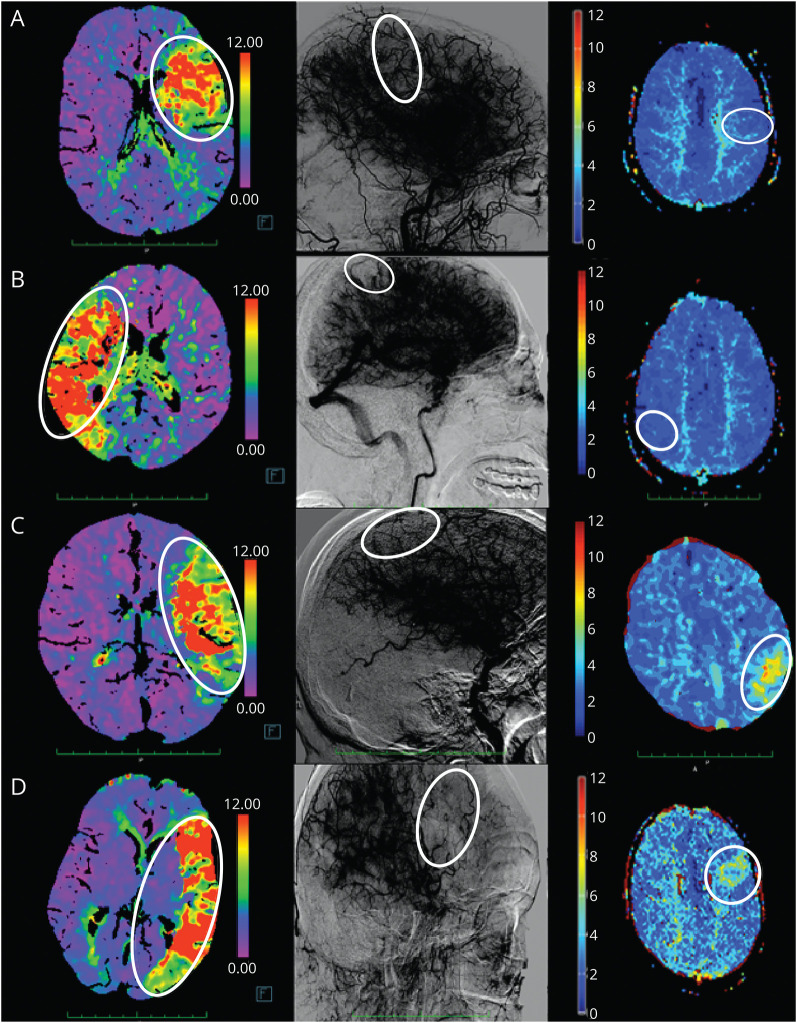
Delayed Reperfusion and Persistent Perfusion Deficit on Follow-Up Perfusion Imaging Runs from final digital subtraction angiography (DSA) images are displayed with high contrast levels to emphasize the capillary phase deficits. Time-to-peak (TTP) perfusion maps were used for these example cases. (A) Admission perfusion imaging shows a left-side M2 occlusion (left panel). DSA at the end of an intervention shows incomplete reperfusion (eTICI 2b67, middle panel). Follow-up perfusion imaging shows complete delayed reperfusion without residual perfusion deficits (right panel). (B) Admission perfusion imaging shows a right-side M1 occlusion (left panel). DSA at the end of an intervention shows incomplete reperfusion (eTICI 2c, middle panel). Follow-up perfusion imaging shows complete delayed reperfusion without residual perfusion deficits (right panel). (C) Admission perfusion imaging shows a left-side M2 occlusion (left panel). DSA at the end of an intervention shows incomplete reperfusion (eTICI 2b67, middle panel). Follow-up perfusion imaging shows persisting perfusion deficit that directly corresponds to the area of incomplete reperfusion from the DSA (right panel). (D) Admission perfusion imaging shows a left-side M1 occlusion (left panel). DSA at the end of an intervention shows incomplete reperfusion (eTICI 2b50, middle panel). Follow-up perfusion imaging shows persisting perfusion deficit that directly corresponds to the area of incomplete reperfusion from the DSA (right panel).

The primary outcome of this target trial was reperfusion status on 24-hour follow-up perfusion imaging maps after incomplete reperfusion (<eTICI 3). This outcome was dichotomized into persistent perfusion deficit (PPD) or DR. PPD refers to a focal, wedge-shaped perfusion delay on follow-up time-to-peak (TTP) or Tmax perfusion imaging maps, corresponding to the capillary phase deficit on the final angiography series. No minimum threshold of hypoperfusion severity or volume was set. DR was defined as the absence of any focal perfusion deficit on follow-up TTP or Tmax perfusion imaging maps, despite incomplete reperfusion on the final angiography series (Figure 1). Because different perfusion imaging software programs were used across the study sites, we evaluated concordance in PPD and DR rating on a random sample of perfusion maps (eFigure 1 and respective caption for methodology). Concordance in rating on different perfusion software programs was reported with the Krippendorf α coefficient (α).

### Descriptive Statistics for Cohort Comparison

Patient demographic and baseline clinical characteristics were extracted from electronic case report forms of randomized trial data and prospective institutional databases. For descriptive summaries, we compared variables of the causal inference analysis among the included cohort to assess heterogeneity. Categorical variables were compared with the Fisher exact test and continuous variables with the Mann-Whitney *U* test. Results are reported as median (interquartile ranges, IQRs) and n (%), unless specified otherwise. IQRs refer to the 25th (Q1) and 75th (Q3) percentile of the distribution. Fixed-effect logistic regression was used to calculate study-specific estimates for baseline occurrence of DR. Afterward, using random intercept adjusted for clustering at the study level, mixed-effect logistic regression was used to account for heterogeneity between the included cohorts and patient characteristics.

### Causal Inference and Target Trial Framework

**Figure 2:**
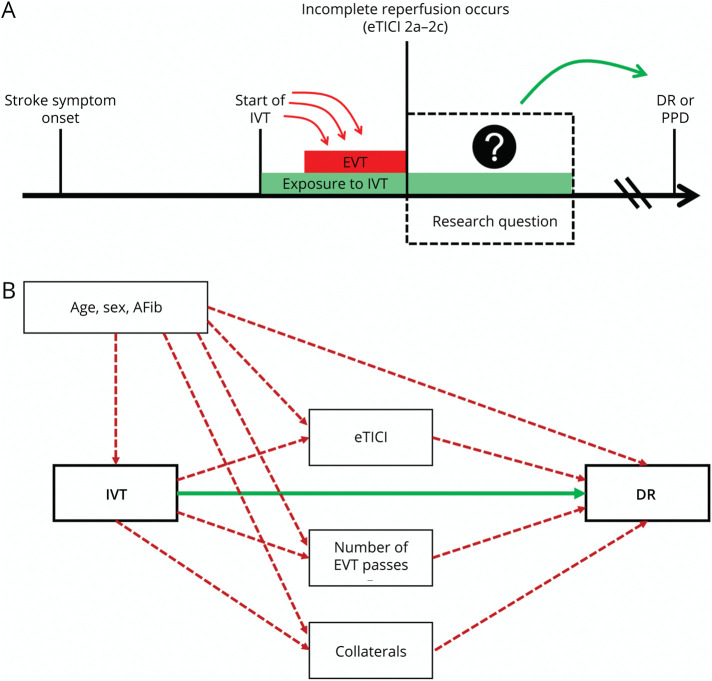
Causal Diagram (A) In a standard patient, IVT is given before the start of EVT. IVT could influence the technical result of EVT including eTICI score, number of passes, and collaterals. After incomplete reperfusion is observed at the end of the EVT, we were interested in whether there was treatment effect of exposure to IVT on DR, given similar age, sex, presence of AFib, eTICI score, number of passes, and collaterals. The research question of this article is related to the remaining thrombolytic treatment effect of already given IVT (dashed square). The primary study hypothesis was that exposure to IVT may increase the chances of having DR at the 24-hour follow-up imaging. (B) The association between IVT and DR is presented through a causal diagram. There are several confounders that may mediate the association of IVT with DR. For example, both eTICI and number of passes are associated with DR and may also be influenced by IVT. On purpose, we closed all causal pathways of IVT by which the occurrence of delayed reperfusion could be influenced indirectly and partially before incomplete reperfusion occurs (eTICI, number of passes, and collaterals). This direct pathway (green line) refers to the effects of IVT that occur after reopening of all the vessels and after EVT has been concluded. In addition, we closed pathways of important confounders that could influence DR but also influence the likelihood of getting IVT (age, sex, AFib). All confounders and mediators are highlighted in red lines. The target trial emulation design allows us to estimate the direct effect of IVT on DR (green line) once a patient experiences incomplete reperfusion, that is, in a given patient with a specific reperfusion score, number of passes, collaterals, etc, does IVT promote DR? AFib = atrial fibrillation; DR = delayed reperfusion; eTICI = expanded Treatment in Cerebral Infarction; EVT = endovascular therapy; IVT = IV thrombolysis; PPD = persistent perfusion deficit.

The primary aim of this target trial emulation was to establish the causal effect between IV thrombolytics and DR on the 24-hour follow-up perfusion imaging, at the time point when a patient experiences incomplete angiographic reperfusion at the end of EVT (Figure 2A). This analysis cannot be used to assess the question whether IVT should be given before EVT, because the total effect of IVT is not calculated and only a subgroup of patients is analyzed (conditional interpretation, i.e., causal effect in only those experiencing incomplete reperfusion, eTICI 2a-2c). However, this analysis can be used to assess a potential residual treatment effect of thrombolytics after EVT, as previously proposed (e.g., administrating intra-arterial lytics or IV thrombolytics after the procedure).^8-10^ We used a causal inference framework to assess whether exposure to IVT was associated with DR on the 24-hour follow-up imaging (Figure 2B). Because the patient population is restricted to patients with eTICI 2a-2c who underwent perfusion imaging at 24 hours, with this target trial emulation, we—on purpose—closed all causal pathways of IVT by which the occurrence of DR could be influenced indirectly.^13,18^ This adjustment of potential mediators allows for the analysis of the direct causal effect that IVT might have on delayed tissue reperfusion. Pathways by which IVT could indirectly influence DR are based on pathophysiologic rationale and previous data showing robust associations with occurrence of DR.^5-7,11^ For example, IVT may influence the final eTICI reperfusion score,^1^ but the main research question is that, given an equal final eTICI score, number of passes, and collateral status, does previous exposure to IVT increases the chances of having DR on 24-hour follow-up imaging? This study does not investigate the immediate effect that IVT might have on preinterventional reperfusion, improved collateral flow, or first-pass effect. Rather, we aim to investigate the delayed effects that IVT might have after EVT has been concluded and the “early” effects of IVT that have already taken place. To isolate the delayed effect of IVT beyond this initial phase, other causal pathways through known mediators were purposely closed off (Figure 2B). This approach does not allow inference of the overall causal effect but instead focuses on the partial effect of IVT on DR in this specific patient population with incomplete reperfusion who underwent perfusion imaging at 24 hours (i.e., conditional interpretation).

To address confounding by indication and selection bias for IVT and allow for causal inference using observational data, we conducted a target trial emulation using 3 parallel methods: (1) G-computations, (2) inverse probability of treatment weighting (IPTW) with propensity scores, and (3) marginal standardization.^13,18^ Details of these methods are described in the Online Supplement (eMethods). The main rationale for the use of 3 different methods within the causal framework was to increase the robustness of our analysis and to show the average causal effect by standardizing the population in different ways without the violation of the positivity assumption or confounding bias.^13,18^ Main in-text results are reported with G-computations with standardized risk differences (sRDs) and standardized risk ratios (sRRs). Results of other methods are given in a table format. Only patients with all available data (complete cases) were included in the final analysis.

### Secondary Analysis

For the prespecified secondary analysis, IVT was stratified according to the type of received lytic (alteplase or tenecteplase) and the treatment effect estimates of these lytics are reported in comparison with the reference category (no lytics). The prespecified secondary analysis included only patients who received IVT. The aim of this secondary analysis was to explore the association between the primary outcome (DR) and 3 different time frames: (1) time elapsed between start of IVT and the end of the intervention (IVT-end-of-EVT); (2) time elapsed between end of the intervention and remaining circulating IVT, based on the terminal half-life of alteplase and tenecteplase of 70 minutes and 90 minutes, respectively (IVT-running-after-EVT); and (3) time of overlap between IVT and EVT (IVT-and-EVT-overlap). All 3 time frames were considered as continuous variables, with sRD and sRR referring to one-hour time. Based on the terminal half-life of thrombolytics, we stratified the latter 2 time frames into quantiles and according to the type of the thrombolytic, as a sensitivity analysis.

Finally, in an exploratory analysis, the benefit of adding IVT to a recently proposed model for predicting DR was assessed with area under the receiver operating characteristic curve (AUC).^6,7^ Two multivariate logistic regression models were constructed with both containing the abovementioned variables, with the exception of one model being adjusted for IVT and the other one not. Performance of the 2 bootstrapped (n = 1,000) AUCs was compared with the DeLong test. All statistical analyses were performed in R statistical software (version 4.3.0).

### Data Availability

Anonymized study data are available from the corresponding author on reasonable request after receipt of a research plan and clearance by the appropriate ethics committee.

## Results

In the end, 832 patients fulfilled the study's inclusion criteria (eFigure 2). The median age was 74 years (IQRs 63–81), 51% (427/832) were male, and 61% (512/832) had DR. There was no substantial heterogeneity between the individual cohorts and centers that were pooled in this study (eTable 2). Concordance in DR rating and PPD between different perfusion software programs was good (α = 0.8, 95% CI 0.7–0.9). When comparing patients with DR and PPD, patients with DR more frequently had atrial fibrillation (35.7% vs 28.4%, *p* = 0.035), lower number of device passes (1 [IQRs 1–2] vs 2 [IQRs 1–3], *p* < 0.001), better collaterals (2 [IQRs 1–3] vs 2 [IQRs 1–2], *p* < 0.001), higher final reperfusion score (for eTICI 2c: 51.6% vs 16.6%, *p* < 0.001), and longer intervention-to-follow-up time (22 hours 42 minutes vs 20 hours 24 minutes, *p* < 0.001). Other characteristics between the groups were comparable (Table 1). After IPTW was performed, there was no substantial difference between the groups (eTable 3).

**Table 1:** Baseline and Interventional Characteristics

	Overall	Delayed reperfusion	Persistent perfusion deficit	*p* Value
N	832	512	320	
Age (median [IQRs])	74 (63, 81)	73 (63, 80)	74 (64, 81)	0.216
Male sex (%)	427 (51.3)	265 (51.8)	162 (50.6)	0.805
Atrial fibrillation (%)	274 (32.9)	183 (35.7)	91 (28.4)	0.035
Device passes (median [IQRs])	2 (1, 2)	1 (1, 2)	2 (1, 3)	<0.001
Intravenous thrombolysis (%)	524 (63.0)	333 (65.0)	191 (59.7)	0.138
ASITN/SIR collateral score (median [IQRs])	2 (1, 3)	2 (1, 3)	2 (1, 2)	<0.001
eTICI (%)				<0.001
2a	54 (6.5)	10 (2.0)	44 (13.8)	
2b50	159 (19.1)	46 (9.0)	113 (35.3)	
2b67	302 (36.3)	192 (37.5)	110 (34.4)	
2c	317 (38.1)	264 (51.6)	53 (16.6)	
Intervention-to-follow-up time in hours (median [IQRs])	21.8 (18.5, 24.5)	22.7 (19.3, 25.3)	20.4 (17.1, 23.2)	<0.001

Abbreviations: ASITN/SIR = American Society of Interventional and Therapeutic Neuroradiology/Society of Interventional Radiology; eTICI = expanded Treatment in Cerebral Infarction; IQR = interquartile range.

Only patients with all available data (complete cases; n = 832) were included in the final analysis (Methods).

**Figure 3:**
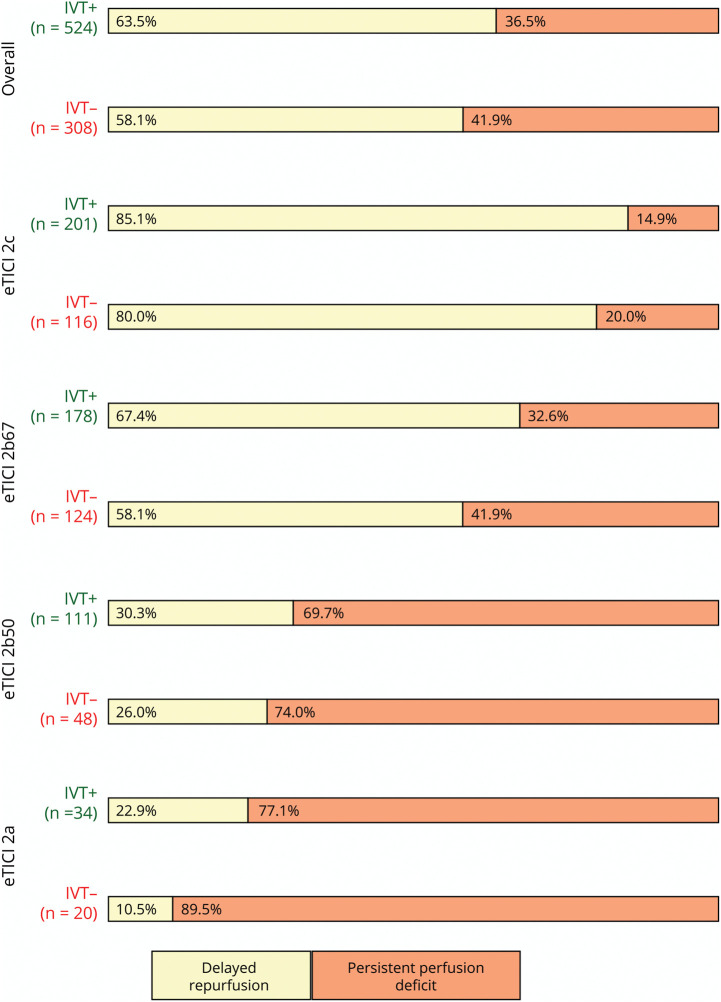
Effect of Intravenous Thrombolytics and Delayed Reperfusion Stratified by the Final Reperfusion Score Bar plots stratified by the incomplete reperfusion score. Percentages refer to the number of patients in each stratum who have either developed persistent perfusion deficit (darker colors) nor delayed reperfusion (brighter colors) at the 24-hour follow-up. Treatment effect of IV thrombolytics on delayed reperfusion was preserved independent of the final reperfusion score (sRR 1.1, 95% CI 1.0–1.3, sRD 8.2%, 95% CI 0.2%–16.1%). eTICI = expanded Treatment in Cerebral Infarction; sRR = standardized risk ratio.

In the main analysis, there was a conditional treatment effect of IVT on the occurrence of DR (sRR 1.1, 95% CI 1.0–1.3, sRD 8.2%, 95% CI 0.2%–16.1%; Figure 3). In addition, patients who have experienced DR had higher likelihood of achieving functional independence (mRS scores 0–2) when compared with patients with PPD (64.2% vs 48.4%, *p* < 0.001). This association was present irrespective of the final eTICI score (eFigure 3). Although patients who have received IVT had higher admission National Institutes of Health Stroke Scale (NIHSS) score, this did not change the conditional treatment effect of IVT on DR (eTable 4). In a prespecified secondary analysis, where IVT was stratified into alteplase and tenecteplase, the direction of the association between DR and IVT remained the same. While the conditional treatment effect of alteplase on DR was no longer present (sRR 1.1, 95% CI 0.9–1.3; sRD 6.7%, 95% CI –1.9% to 15.5%; Table 2), conditional effect of tenecteplase on DR was preserved (sRR 1.2, 95% CI 1.0–1.4, sRD 10.7%, 95% 1.0%–20.4%). When tenecteplase was stratified according to different doses, the treatment effect was only preserved in the 0.25-mg/kg dose range (eTable 5). Rates of symptomatic intracranial hemorrhage were comparable between different thrombolytics (eTable 6).

**Table 2:** Target Trial Emulation

Methods						
	G-computations^a^		Inverse probability of treatment weighting^a^		Marginal standardization^a^	
	sRR (95% CI)	sRD (95% CI)	sRR (95% CI)	sRD (95% CI)	sRR (95% CI)	sRD (95% CI)
Primary analysis						
Intravenous thrombolysis	1.13 (1.02–1.28)	8.2 (0.2–16.1)	1.10 (1.03–1.17)	6.0 (2.1–9.8)	1.11 (1.02–1.23)	7.3 (0.2–12.2)
Secondary analysis						
Type of lytic						
Alteplase	1.11 (0.96–1.27)	6.7 (−1.9–15.5)	1.08 (1.00–1.18)	5.2 (−0.5–9.7)	1.09 (0.97–1.22)	5.1 (−1.4–11.6)
Tenecteplase	1.17 (1.01–1.36)	10.7 (1.0–20.4)	1.11 (1.03–1.20)	7.6 (2.1–11.2)	1.14 (1.01–1.29)	8.2 (7.48–15.66)
IVT-end-of-EVT time in hours^b^	0.93 (0.87–0.98)	−5.2 (−9.1 to −1.3)	0.95 (0.93–0.99)	−3.1 (−8.5 to −2.6)	0.94 (0.90–0.98)	−3.9 (−9.7 to −1.4)

Abbreviations: IVT = IV thrombolysis; sRD = standardized risk difference; sRR = standardized risk ratio.

a Logistic regression models were adjusted for age, sex, atrial fibrillation, device passes, collateral score, final reperfusion score, and time from the end of an intervention until the next follow-up.

b Only patients who have received IVT (n = 524/832) were included in this analysis.

In a subanalysis of patients who received IVT (n = 524/832), we observed findings comparable with the ones in the main analysis when stratifying the patients based on perfusion imaging outcome (DR vs PPD, eTables 7 and 8). Among patients who have received IVT, there was a causal effect of shorter time between IVT start and end of the intervention on the occurrence of DR (sRR 0.93, 95% CI 0.87–0.98; sRD −5.2%, 95% CI −9.1% to −1.3% per hour increase, eFigure 4) and higher likelihood of DR if there was remaining circulating IVT at the end of the intervention (sRR 1.12, 95% CI 1.07–1.18; sRD 8.1%, 95% CI 4.4%–11.7% per hour increase, eTable 9). There was no causal effect between the IVT-and-EVT-overlap time and DR. A more pronounced effect of IVT-running-after-EVT time was seen among patients in the tenecteplase group across all quantiles (eTable 10) while IVT-and-EVT-overlap time remained not associated with DR, irrespective of the time quantile of choice of lytic (eTable 11).

When IVT was included together with other variables that are known to predict DR,^6,7^ there was no difference in the predictive accuracy of the model with and without IVT (AUC 0.82, 95% CI 0.79–0.82 vs AUC 0.81, 95% CI 0.78–0.81; *p* = 0.08). Comparable results were observed when IVT was stratified into alteplase and tenecteplase and with the model without IVT (eFigure 5).

## Discussion

In our pooled international multicenter data set comprising 12 comprehensive thrombectomy centers across Europe, Australia, and New Zealand, we observed the following: (1) Exposure to thrombolytics showed conditional treatment effect on the occurrence of delayed complete reperfusion despite incomplete reperfusion after thrombectomy. (2) The treatment effect of thrombolytics on DR seemed to be stronger if the time between start of IV thrombolytics and the occurrence of incomplete reperfusion was short or if there were higher chances of circulating lytics at the end of the intervention. (3) Although there was no significant difference between alteplase and tenecteplase in achieving DR, point estimates suggest a potentially stronger effect for tenecteplase.

Observational comparisons have suggested a potential benefit of preinterventional IVT^19^ while randomized controlled trials of directly admitted patients receiving EVT + IVT or EVT alone could not confirm this hypothesis,^1^ except in the early time window (2h 30min).^20^ This discrepancy is likely attributable to the indication bias from observational studies that makes it difficult to estimate the clinical effect of IVT. Particularly, IVT-ineligible patients can present with factors that predispose them to poorer outcome.^21^ Instead of focusing purely on clinical outcomes, we wanted to highlight a potential mechanism by which IVT may promote reperfusion. In the Improving Reperfusion Strategies in Ischemic Stroke (IRIS) collaboration, patients allocated to EVT + IVT had higher rates of successful reperfusion compared with patients allocated to EVT alone.^1^ In this study, preinterventional IVT showed conditional treatment effect on complete DR with the resolution of distal emboli and higher likelihood of better clinical outcome. The likelihood for better outcome was present irrespective of the final eTICI grade. The target trial approach enabled us to estimate conditional causality of IVT on incomplete reperfusion in a pooled data set of both real-world and trial patients. The robustness of our findings is supported by the preserved effect throughout different methods in the target trial emulation.^13,18^ Previous trials have reported on the effect of IVT on clinical outcome at 3 months while data on tissue reperfusion, as assessed on the perfusion imaging at 24-hour follow-up, are not available. Therefore, it could be possible that a subset of patients in the original trials could have experienced DR due to IVT and contributed to the higher rates of favorable outcome at 3 months, especially if IVT was received early after stroke symptom onset (<2h 30min).^20^ This also suggests that a reperfusion-favoring effect of added IVT may not be limited to the final angiographic results. This is particularly relevant because incomplete reperfusion is one important, potentially modifiable, factor that limits the benefit of EVT.^2-4^ This analysis also suggests that a potential benefit of IVT in the setting of EVT may be particularly high among patients in whom remaining distal vessel occlusions persist or occur after EVT. Ideally, both early (during the intervention) and late (24-hour follow-up) reperfusion should be evaluated on the same imaging modality, that is, perfusion imaging. Flat-panel perfusion imaging has already been proposed to complement the evaluation of reperfusion efficacy in the angiography suite.^22^ This imaging modality can be easily integrated into the interventional workflow and does not require patient movement. Serial acquisition of flat-panel perfusion imaging might provide critical information on tissue reperfusion while the patient is in the operating suite and provide further evidence on the temporal evolution of incomplete reperfusion.^23^

The terminal half-life of alteplase and tenecteplase is 70 minutes and 90 minutes, respectively.^24^ Therefore, it is biologically plausible that we observed a stronger conditional effect with DR in IVT-treated patients if the time between administration of IVT and occurrence of incomplete reperfusion is short. While we cannot make definite conclusions about causality, it seems to be a likely explanation that circulating alteplase and tenecteplase after thrombectomy promotes clearance of remaining vessel occlusion by fibrinolysis.^25^ The beneficial effect of additional alteplase among patients with incomplete reperfusion has previously been demonstrated by the CHOICE trial.^26^ Although the time points of lytic administration were different in our study when compared with the CHOICE trial (before the intervention vs after incomplete reperfusion was seen on angiography, respectively), the mechanisms by which thrombolytics promote clearance of remaining occlusions should be the same. However, the CHOICE trial could not demonstrate this benefit of thrombolytics among patients with eTICI 2b50/67 vs eTICI 2c/3 reperfusion (*p* > 0.5).^26^ Future efforts from the IRIS collaboration may evaluate the effect of thrombolytics on DR within each eTICI stratum to provide additional data.

This study does not investigate whether thrombolytics should be given before EVT. Therefore, our results currently do not allow for individualized decision making on giving or withholding IVT. Instead, we investigate the effects of circulating thrombolytics on residual occlusions after EVT among patients with incomplete reperfusion who undergo perfusion imaging at the 24-hour follow-up. Patients with remaining circulating thrombolytics at the conclusion of the intervention with distal occlusions had a higher likelihood of spontaneous clearance of these occlusions because of the continued activity of the circulating thrombolytics, especially among patients receiving tenecteplase. Previous studies have demonstrated the reperfusion-favoring effect of thrombolytic before EVT^16,27^ while other studies have demonstrated this effect of thrombolytics after EVT (CHOICE, NCT03876119; Intra-arterial tenecteplase after successful endovascular therapy [ANGEL TNK], NCT05624190; Intra-arterial alteplase for acute ischemic stroke after mechanical thrombectomy [PEARL], NCT05856851, presented at the International Stroke Conference, February 5–7 2025, Los Angeles, CA). Bridging this gap (thrombolytics before vs after EVT) are studies showing benefit of complementing and/or continuing with thrombolytics in settings of failed/unsuccessful EVT.^8,9^

Consistent with previous reports, we also underline potential therapeutic effect of thrombolytics in the treatment of patients with incomplete reperfusion. This effect is presently being investigated by several randomized controlled trials (TECNO, NCT05499832; CHOICE II, NCT05797792; RESCUE-TNK, NCT05657470), and their results will provide further information on potential benefits of thrombolytics in cases of incomplete reperfusion. Although the mode of lytic administration differs between these trials and our study (intra-arterial vs intravenous, respectively), it should not affect the mechanism of thrombolytics on residual occlusions. The reperfusion-favoring effect of IVT is further supported by pathophysiologic reasoning, as thrombolytics are anticipated to attenuate or dissolve thrombi, regardless of the timing of administration. Moreover, we would expect a stronger treatment effect of intra-arterially given lytics because of higher concentration on a local level. If the present results are validated externally, thrombolytics may be administrated more liberally if the chances of incomplete reperfusion are suspected to be high^6,7^ and concepts such as reverse bridging therapy should be tested.

Although there was no conditional treatment effect between administering alteplase and achieving DR, the point estimates suggested a stronger effect with tenecteplase. A similar effect was observed when evaluating the remaining circulating IVT after EVT, whereas only tenecteplase remained associated with DR. Previous trials have compared the immediate effect of alteplase and tenecteplase on initial reperfusion rates and have shown a benefit of tenecteplase for achieving early successful reperfusion.^15,16^ We have evaluated the delayed effects of tenecteplase on the 24-hour perfusion imaging among EVT-treated patients, and our results are in line with previous studies on the effects of tenecteplase in the immediate acute and peri-interventional setting.^15,16^ Potential explanations may be attributed to the longer terminal half-life of tenecteplase, different pharmacokinetics, and generally shorter time delays with the novel thrombolytic (onset-to-puncture, onset-to-reperfusion, puncture-to-reperfusion, etc).^28^ Treatment effect of tenecteplase was preserved in the 0.25 mg/kg stratum, which seems consistent with the previous studies on the effectiveness of tenecteplase and recent recommendations.^16,29^ It is important to note that we saw no major safety concerns irrespective of the choice or dose of the lytic. Having information on preinterventional IVT could be useful when predicting the occurrence of DR on follow-up imaging.^6,7^ Although the difference in the AUCs was not statistically significant, there was an increase in the predictive benefit for DR when IVT was added. It should be underlined that statistical significance merely indicates an association between 2 variables. This, however, does not guarantee on the magnitude of this association nor on the underlying distribution of the variable for given outcomes.^30^ Complex interactions or confounding factors might exist beyond what significance alone can identify.^30^

This is a retrospective observational study cohort and is subject to inherent study design–related biases. In such settings, target trial emulation is a recommended approach to estimate causality and treatment effects of a given intervention.^13,18^ This study pooled the data of 3 study cohorts coming from different sources; however, there was no substantial heterogeneity between the cohorts, allowing us to treat the pooled data set as a single cohort for study purposes. The number of participants who have undergone perfusion imaging across the studies was different: EXTEND-IA: 11/34 (32.4%); EXTEND-IA TNK part 1: 134/152 (88.2%); EXTEND-IA TNK part 2: 167/185 (90.3%); Graz: 146/170 (85.9%); Bern: 518/914 (56.7%). Patients who undergo perfusion imaging at 24 hours tend to have better clinical presentation at baseline compared with those not undergoing perfusion imaging at follow-up.^5-7^ Decision to undergo perfusion imaging is dependent on the study, and some studies included only IVT patients, which could have introduced further bias. Owing to this, we cannot infer absolute numbers of DR nor the direction of potential association between DR and IVT among patients who did not undergo perfusion imaging at 24 hours. Consequently, these results allow for only conditional, rather than overall, interpretation of causality between IVT and DR, and these findings cannot be used to estimate the overall causal effect. Only variables that have been previously described^5,11,12^ to be associated with the evolution of incomplete reperfusion were considered for this analysis, and whether resulting adjustments appropriately compensated for residual confounding remains uncertain. Although we calculated the effects of alteplase and tenecteplase based on their terminal half-life pharmacokinetics, the different mode of administration—infusion for alteplase and bolus for tenecteplase—introduces uncertainty regarding the actual duration and their effect. Time from symptom onset until start of IV thrombolysis could also be considered to investigate the effect of IV thrombolysis on DR. However, this timing was not available for all patients, nor have all patients received IV thrombolysis.

Exposure to thrombolytics showed independent treatment effect on the occurrence of DR among patients with incomplete reperfusion at the end of an intervention who undergo perfusion imaging at the 24-hour follow-up. The effect of thrombolytics on DR was observed only if there was a high chance of therapeutic concentrations of thrombolytics at the time point when the proximal vessel was recanalized, but distal occlusions persisted and/or occurred.

## Glossary

AUC: area under the receiver operating characteristic curve
DR: delayed reperfusion
eTICI: expanded Treatment in Cerebral Infarction
EVT: endovascular thrombectomy
IPTW: inverse probability of treatment weighting
IRIS: Improving Reperfusion Strategies in Ischemic Stroke
IVT: intravenous thrombolysis
PPD: persistent perfusion deficit
sRD: standardized risk difference
sRR: standardized risk ratio
TTP: time-to-peak

## References

[1] Majoie CB, Cavalcante F, Gralla J, et al. Value of intravenous thrombolysis in endovascular treatment for large-vessel anterior circulation stroke: individual participant data meta-analysis of six randomised trials. Lancet. 2023;402(10406):965-974. doi:10.1016/s0140-6736(23)01142-x 37640037

[2] Powers WJ, Rabinstein AA, Ackerson T, et al. Guidelines for the early management of patients with acute ischemic stroke: 2019 update to the 2018 guidelines for the early management of acute ischemic stroke a guideline for healthcare professionals from the American Heart association/American stroke association. Stroke. 2019;50(12):e344-e418. doi:10.1161/STR.0000000000000211 31662037

[3] Berge E, Whiteley W, Audebert H, et al. European Stroke Organisation (ESO) guidelines on intravenous thrombolysis for acute ischaemic stroke, Eur stroke J. 2021;6(1):I-LXII. doi:10.1177/2396987321989865 PMC7995316 33817340

[4] LeCouffe NE, Kappelhof M, Treurniet KM, et al. 2B, 2C, or 3. Stroke. 2020;51(6):1790-1796. doi:10.1161/STROKEAHA.119.028891 32397926

[5] Mujanovic A, Jungi N, Kurmann CC, et al. Importance of delayed reperfusions in patients with incomplete thrombectomy. Stroke. 2022;53(11):3350-3358. doi:10.1161/STROKEAHA.122.040063 36205143 PMC9586830

[6] Mujanovic A, Brigger R, Kurmann CC, et al. Prediction of delayed reperfusion in patients with incomplete reperfusion following thrombectomy. Eur Stroke J. 2023;8(2):456-466. doi:10.1177/23969873231164274 37231686 PMC10334170

[7] Mujanovic A, Ng FC, Branca M, et al. External validation of a model for persistent perfusion deficit in patients with incomplete reperfusion after thrombectomy: EXTEND-PROCEED. Neurology. 2024;103(2):e209401. doi:10.1212/WNL.0000000000209401 38900979 PMC11254450

[8] Weller JM, Dorn F, Petzold GC, Bode FJ., GSR-ET investigators. Intravenous thrombolysis upon flow restoration improves outcome in endovascular thrombectomy. J Neurointerv Surg. 2023;15(e2):E229-E231. doi:10.1136/jnis-2022-019522 36307203 PMC10646906

[9] Burian E, Sepp D, Lehm M, et al. Start, stop, continue? The benefit of overlapping intravenous thrombolysis and mechanical thrombectomy: a matched case-control analysis from the German stroke registry. Clin Neuroradiol. 2023;33(1):187-197. doi:10.1007/s00062-022-01200-y 35881162 PMC10014683

[10] Laredo C, Rodríguez A, Oleaga L, et al. Adjunct thrombolysis enhances brain reperfusion following successful thrombectomy. Ann Neurol. 2022;92(5):860-870. doi:10.1002/ana.26474 36054449 PMC9804472

[11] Mujanovic A, Kammer C, Kurmann CC, et al. Association of intravenous thrombolysis with delayed reperfusion after incomplete mechanical thrombectomy. Clin Neuroradiol. 2023;33(1):87-98. doi:10.1007/s00062-022-01186-7 35833948 PMC10014807

[12] Rubiera M, Garcia-Tornel A, Olivé-Gadea M, et al. Computed tomography perfusion after thrombectomy: an immediate surrogate marker of outcome after recanalization in acute stroke. Stroke. 2020;51(6):1736-1742. doi:10.1161/STROKEAHA.120.029212 32404034

[13] Hernán MA, Wang W, Leaf DE. Target trial emulation: a framework for causal inference from observational data. JAMA. 2022;328(24):2446-2447. doi:10.1001/jama.2022.21383 36508210

[14] Campbell BCV, Mitchell PJ, Kleinig TJ, et al. Endovascular therapy for ischemic stroke with perfusion-imaging selection. N Engl J Med. 2015;372(11):1009-1018. doi:10.1056/nejmoa1414792 25671797

[15] Campbell BCV, Mitchell PJ, Churilov L, et al. Tenecteplase versus alteplase before thrombectomy for ischemic stroke. N Engl J Med. 2018;378(17):1573-1582. doi:10.1056/nejmoa1716405 29694815

[16] Campbell BCV, Mitchell PJ, Churilov L, et al. Effect of intravenous tenecteplase dose on cerebral reperfusion before thrombectomy in patients with large vessel occlusion ischemic stroke: the EXTEND-IA TNK Part 2 randomized clinical trial. JAMA. 2020;323(13):1257-1265. doi:10.1001/jama.2020.1511 32078683 PMC7139271

[17] Kneihsl M, Hinteregger N, Nistl O, et al. Post-reperfusion hyperperfusion after endovascular stroke treatment: a prospective comparative study of TCD versus MRI. J Neurointerv Surg. 2023;15(10):983-988. doi:10.1136/jnis-2022-019213 36137745

[18] Robins JM, Miguel Angel H, Babetter B. Marginal structural models and causal inference in Epidemiology. Epidemiology. 2000;5(11):550-560. doi:10.1097/00001648-200009000-00011 10955408

[19] Mistry EA, Mistry AM, Nakawah MO, et al. Mechanical thrombectomy outcomes with and without intravenous thrombolysis in stroke patients: a meta-analysis. Stroke. 2017;48(9):2450-2456. doi:10.1161/STROKEAHA.117.017320 28747462

[20] Kaesmacher J, Cavalcante F, Kappelhof M, et al. Time to treatment with intravenous thrombolysis before thrombectomy and functional outcomes in acute ischemic stroke A meta-analysis. JAMA. 2024;331(9):764-777. doi:10.1001/jama.2024.0589 38324409 PMC10851137

[21] Kaesmacher J, Mordasini P, Arnold M, et al. Direct mechanical thrombectomy in tPA-ineligible and-eligible patients versus the bridging approach: a meta-analysis. J Neurointerv Surg. 2019;11(1):20-27. doi:10.1136/neurintsurg-2018-013834 29705773 PMC6327861

[22] Mujanovic A, Kurmann CC, Manhart M, et al. Value of immediate flat panel perfusion imaging after endovascular therapy (AFTERMATH): a proof of concept study. AJNR Am J Neuroradiol. 2024;45(2):163-170. doi:10.3174/ajnr.A8103 38238089 PMC11285981

[23] Mujanovic A, Windecker D, Cimflova P, et al. Natural evolution of incomplete reperfusion in patients following endovascular therapy after ischemic stroke. Stroke. 2025;56(2):447-455. doi:10.1161/STROKEAHA.124.049641 39567366 PMC11771359

[24] Logallo N, Kvistad CE, Thomassen L. Therapeutic potential of tenecteplase in the management of acute ischemic stroke. CNS Drugs. 2015;29(10):811-818. doi:10.1007/s40263-015-0280-9 26387127

[25] Desilles JP, Loyau S, Syvannarath V, et al. Alteplase reduces downstream microvascular thrombosis and improves the benefit of large artery recanalization in stroke. Stroke. 2015;46(11):3241-3248. doi:10.1161/STROKEAHA.115.010721 26443832

[26] Renú A, Millán M, San Román L, et al. Effect of intra-arterial alteplase vs placebo following successful thrombectomy on functional outcomes in patients with large vessel occlusion acute ischemic stroke: the CHOICE randomized clinical trial. JAMA. 2022;327(9):826-835. doi:10.1001/jama.2022.1645 35143603 PMC8832304

[27] Mujanovic A, Eker O, Marnat G, et al. Association of intravenous thrombolysis and pre-interventional reperfusion: a post hoc analysis of the SWIFT DIRECT trial. J Neurointerv Surg. 2023;15(e2):e232-e239. doi:10.1136/jnis-2022-019585 36396433 PMC10646907

[28] Warach SJ, Dula AN, Milling TJ. Tenecteplase thrombolysis for acute ischemic stroke. Stroke. 2020;51(11):3440-3451. doi:10.1161/STROKEAHA.120.029749 33045929 PMC7606819

[29] Alamowitch S, Turc G, Palaiodimou L, et al. European Stroke Organisation (ESO) expedited recommendation on tenecteplase for acute ischaemic stroke. Eur Stroke J. 2023;8(1):8-54. doi:10.1177/23969873221150022 37021186 PMC10069183

[30] Lo A, Chernoff H, Zheng T, Lo SH. Why significant variables aren't automatically good predictors. Proc Natl Acad Sci U S A. 2015;112(45):13892-13897. doi:10.1073/pnas.1518285112 26504198 PMC4653162

